# Association of Sarcopenia With Global, Motor, Cognitive, and Swallowing Function Prognosis in Stroke Patients: A Systematic Review and Meta‐Analysis

**DOI:** 10.1002/brb3.71810

**Published:** 2026-09-29

**Authors:** Dandan Xie, Mingming Ma, Lu Chen

**Affiliations:** ^1^ Department of Rehabilitation Hangzhou Red Cross Hospital Hangzhou Zhejiang China; ^2^ Department of Neurology Hangzhou Red Cross Hospital Hangzhou Zhejiang China

**Keywords:** functional prognosis, meta‐analysis, sarcopenia, stroke, systematic review

## Abstract

**Objective:**

Sarcopenia has been demonstrated to correlate with the prognosis of stroke. This study aimed to systematically evaluate the impact of sarcopenia on global, motor, cognitive, and swallowing function outcomes in stroke patients.

**Methods:**

Relevant studies investigating the association between sarcopenia and multidimensional prognostic outcomes in stroke were retrieved from the PubMed, Embase, Web of Science, Cochrane Library, and Scopus databases. The primary outcome was the risk of poor global functional prognosis assessed by the modified Rankin Scale (mRS). Secondary outcomes included motor function scores (Functional Independence Measure‐motor, FIM‐motor), cognitive function scores, and the risk of swallowing dysfunction.

**Results:**

A total of 36 cohort studies were finally included. Pooled results indicated that stroke patients with comorbid sarcopenia faced a notably elevated risk of poor global functional prognosis (mRS, OR = 2.43, 95% CI: 1.95 to 3.03, *p* < 0.001). The motor function scores (FIM‐motor, SMD = −0.94, 95% CI: −1.11 to −0.77, *p* < 0.001) and cognitive function scores (FIM‐cognition or Montreal Cognitive Assessment, SMD = −0.95, 95% CI: −1.22 to −0.69, *p* < 0.001) in the sarcopenia group were significantly lower than those in the non‐sarcopenia group, while the risk of swallowing dysfunction showed a notable but non‐significant elevation (endotracheal intubation or Functional Oral Intake Scale, OR = 1.45, 95% CI: 0.94 to 2.25, *p* = 0.09). Subgroup analyses indicated that the patient populations, cut‐off value of mRS, follow‐up duration, stroke type, and diagnostic criteria for sarcopenia were the main sources of heterogeneity. Sensitivity analyses confirmed the robustness of the results. Egger's test suggested the presence of publication bias, but the conclusions remained valid after correction using the trim‐and‐fill method.

**Conclusion:**

Sarcopenia is an important risk factor for poor global, motor, cognitive, and swallowing function prognosis in stroke patients. In clinical practice, sarcopenia screening should be incorporated into the routine assessment of stroke patients, and individualized interventions should be promptly implemented for high‐risk populations to improve long‐term prognosis.

## Graphical abstract

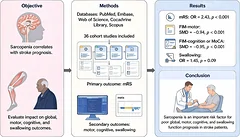

## 1 Introduction

Stroke ranks among the leading causes of mortality and long‐term disability worldwide, imposing a substantial burden on patients' families and the healthcare system due to its high incidence and disability rates (Saini et al. 2021). With advances in medical technology, the survival rate of stroke patients in the acute phase has improved significantly, yet there exists considerable variability in long‐term functional recovery. Approximately 30%‐60% of patients experience residual impairments, including motor dysfunction, cognitive impairment, and dysphagia, which severely compromise their ability to live independently and their quality of life (Jiang et al. 2025; Tang et al. 2025; Uhrin et al. 2025). Therefore, identifying the key influencing factors of post‐stroke functional prognosis is of great clinical and public health significance for formulating individualized rehabilitation strategies and improving long‐term outcomes.

Sarcopenia is a geriatric syndrome characterized primarily by the loss of skeletal muscle mass, decreased muscle strength, and impaired muscle function. Its pathogenesis is associated with multiple factors such as aging, nutritional disorders, and abnormal neuromuscular regulation (Evans 2010). In recent years, studies have found that stroke patients exhibit a 24% reduction in volume of skeletal muscle in the paralyzed side (Ryan et al. 2011), and the prevalence of sarcopenia in stroke patients is approximately 40% (Dos Santos et al. 2026). Moreover, sarcopenia is closely correlated with post‐stroke functional impairments, which may be attributed to post‐stroke denervation, proteolysis induced by stroke‐related inflammation and oxidative stress, and disuse atrophy (Li et al. 2020; Su et al. 2020). Theoretically, sarcopenia may affect stroke prognosis through multiple pathways. Firstly, skeletal muscle serves as the core carrier of motor function in the body and the decline in its quantity and quality directly leads to impaired motor capacity, increasing the risk of falls and activity limitations (Feng et al. 2024). Secondly, muscle tissue acts as an important endocrine organ that can secrete myokines, like irisin and brain‐derived neurotrophic factor (BDNF), to participate in neural repair and metabolic regulation. Sarcopenia may exacerbate neurological damage by reducing the release of these protective factors, thereby impairing multiple system functions such as cognition and swallowing (Han et al. 2025). In addition, sarcopenia interacts with issues like malnutrition, inflammatory responses, and reduced rehabilitation compliance in stroke patients, which further elevates the risk of adverse outcomes (Yoshimura et al. 2019).

Although several studies have explored the association between sarcopenia and stroke prognosis, the existing evidence still has many limitations. Most studies have focused on a single functional outcome, such as hospital stay duration and overall neurological function, lacking a comprehensive analysis of multidimensional outcomes including motor function, cognitive function, and swallowing function (Gheri et al. 2024; Li et al. 2023; Yang et al. 2025). Although the study by Yang et al. (2025) described the impact of sarcopenia on multidimensional post‐stroke outcomes, no quantitative data analysis was performed. Furthermore, the sample sizes of existing studies vary greatly. Some small‐sample studies may have result bias, and the sources of heterogeneity remain unclear. Therefore, it is necessary to quantitatively evaluate the impact of sarcopenia on multiple aspects of outcomes in stroke patients, clarify the strength of its role as a prognostic risk factor, and explore the sources of heterogeneity, so as to provide high‐quality evidence‐based medical evidence for the early intervention and rehabilitation management of stroke patients in clinical practice.

Based on the above, this systematic review and meta‐analysis was conducted to address the following research questions: (1) Does sarcopenia increase the risk of poor long‐term global functional prognosis assessed by mRS scores in stroke patients? (2) Does sarcopenia exert adverse effects on motor function, cognitive function, and swallowing function in stroke patients? (3) Does the association between sarcopenia and stroke prognosis vary across different subgroups, such as different diagnostic criteria for sarcopenia, different follow‐up durations, and different stroke types? The findings of this study will offer important insights for optimizing post‐stroke rehabilitation strategies.

## 2 Methods

Guided by the Preferred Reporting Items for Systematic Reviews and Meta‐Analyses (PRISMA) statement and the Cochrane Collaboration's methodological suggestions, the study was carried out in strict compliance with standardized reporting requirements, and its protocol has been registered on the International Prospective Register of Systematic Reviews (PROSPERO, registration number: CRD420251248300).

### 2.1 Search Strategy

A total of five databases were searched, including PubMed, Embase, Web of Science, the Cochrane Library, and Scopus, from their inception to December 21, 2025. The search keywords included “stroke,” “cerebrovascular accident,” “cerebrovascular disorders,” “cerebral infarction,” “cerebral hemorrhage,” “cerebrovascular infarction,” “intracranial hemorrhage,” “sarcopenia,” “muscle loss,” “muscular atrophy,” “muscle weakness,” “muscle atrophy,” “muscle wasting,” “muscle mass,” “muscle index,” “sarcopenic,” “sarcopaenic,” and “sarcopaenia.” A comprehensive breakdown of the search strategy is presented in Table S1.

### 2.2 Selection Criteria

We established the following inclusion criteria: (i) study type: cohort study; (ii) study population: patients with definitively diagnosed stroke, including ischemic, hemorrhagic, or mixed stroke; (iii) at the start of the study, the stroke population was divided into sarcopenia (suspected sarcopenia or pre‐sarcopenia) and non‐sarcopenia groups, with sarcopenia diagnosed by the Asian Working Group for Sarcopenia (AWGS), the European Working Group on Sarcopenia in Older People (EWGSOP) or other methods; (iv) study indicators including at least one of the following prognostic outcomes of stroke: global functional outcome assessed by the modified Rankin Scale (mRS); motor function assessed by the Functional Independence Measure‐motor (FIM‐motor) subscale; cognitive function assessed by validated cognitive screening instruments, such as FIM‐cognition, Montreal Cognitive Assessment (MoCA), or Mini‐Mental State Examination (MMSE); and swallowing function assessed by validated dysphagia assessment tools, such as nasogastric tube placement, Functional Oral Intake Scale (FOIS), or water swallow test (WST).

The exclusion criteria were as follows: (i) incomplete data; (ii) statistical form of outcome indicators not meeting the requirements; (iii) small sample size (less than 10 cases per group).

### 2.3 Outcome Indicators

The primary outcome was the impact of sarcopenia on the global functional prognosis of stroke patients, assessed by mRS. The mRS is a widely used 7‐point ordinal scale (ranging from 0 to 6) that measures the degree of disability or dependence in activities of daily living (ADL) following stroke. Higher scores indicate greater disability: 0 = no symptoms, 1 = no significant disability despite symptoms, 2 = slight disability (unable to carry out all previous activities but able to look after own affairs without assistance), 3 = moderate disability (requires some help but able to walk without assistance), 4 = moderately severe disability (unable to walk or attend to bodily needs without assistance), 5 = severe disability (bedridden and requiring constant nursing care), and 6 = death. Poor global functional outcome was defined according to each original study, typically as mRS scores ≥ 2, ≥ 3, or ≥ 4, and the specific cutoff value used in each study was retained for analysis. Effect size was expressed as odds ratio (OR) with 95% confidence interval (CI).

There were three secondary outcomes, including the impact of sarcopenia on motor function, cognitive function, and swallowing function in stroke patients. Specifically, motor function was assessed using FIM‐motor subscale, which covers motor‐related domains including self‐care, sphincter control, transfers, and locomotion, with lower scores indicating greater motor dependence. Regarding cognitive function, it was assessed primarily using FIM‐cognition subscale, the MoCA or the MMSE. FIM‐cognition evaluates comprehension, expression, social interaction, problem‐solving, and memory. The MoCA assesses attention, executive function, memory, language, and visuospatial skills. The MMSE assesses orientation, registration, attention and calculation, recall, and language. Lower scores on any of these measures indicate poorer cognitive function. As for swallowing dysfunction, it was assessed according to the definitions used in each original study, including the placement of a nasogastric tube, FOIS score < 5, or WST ≥ 3. Effect size was expressed as standardized mean difference (SMD) or OR, with their corresponding 95% CI.

### 2.4 Data Extraction

Literature eligibility assessment and data abstraction were conducted in duplicate by two reviewers. All inconsistencies were addressed via group consensus discussions. Information extracted from each selected study included the following: first author, year of publication, study country, sample size, age, gender, stroke type, sarcopenia diagnostic method, outcome indicators, and follow‐up duration.

### 2.5 Quality Evaluation

The Newcastle–Ottawa Quality Assessment Scale (NOS) was used for quality evaluation. The assessment included three main domains: selection (representativeness of the exposed cohort, selection of the non‐exposed cohort, assessment of exposure, demonstration that outcome of interest was not present at the start), comparability (control for important factors and additional factors), and outcome (outcome assessment, follow‐up long enough for outcome to occur, adequacy of follow‐up). Each item was scored 1 point, with a total score ranging from 1 to 9. Studies with a score of 7–9 were considered high quality, 5–6 as moderate quality, and 1–4 as low quality.

### 2.6 Statistical Analysis

Meta‐analysis was performed using RevMan 5.3 software (Cochrane Collaboration, Copenhagen, Denmark) and Stata 16.0 software (StataCorp LLC, College Station, TX, USA). For dichotomous outcomes, OR with 95% CI was used as the effect size. To ensure consistency in the direction of effect sizes, all effect sizes from the included studies were unified to reflect the association direction of “sarcopenia leading to adverse outcomes in stroke.” If the original study directly reported an OR value with an inconsistent direction, the reciprocal transformation was applied. The hazard ratio (HR) for short‐term outcomes (≤ 6 months) was approximated as the OR value. A transformed OR > 1 indicated that sarcopenia increased the risk of adverse outcomes. For continuous outcomes, SMD with 95% CI was used as the effect size. For some continuous data reported as median with interquartile range [*M* (Q1, Q3)], if the data approximately followed a normal distribution, the mean (x̄) was set equal to *M* and the standard deviation (SD) was calculated as (Q3–Q1)/1.35, whereas if the data showed a significantly skewed distribution, the study was excluded. Cochran's *Q* test was applied to quantify heterogeneity. Values of *p* < 0.1 and *I* ^2^ > 50% were considered indicative of high heterogeneity and a random‐effects model was adopted. Subgroup analyses were carried out focusing on the main outcome to explore the factors contributing to heterogeneity. One‐study removal method was used for sensitivity analysis to examine the robustness of the results to individual studies. Publication bias was evaluated using Egger's test and visually presented with a funnel plot, and the trim‐and‐fill method was conducted to address publication bias. *p* < 0.05 was considered statistically significant.

## 3 Results

### 3.1 Literature Search Results

Totally, 4842 relevant studies were retrieved from the five databases, of which 2363 were duplicates, leaving 2479 studies for title and abstract screening. Among these, 113 were non‐human studies, 677 were non‐observational studies, 121 were not in the article format, and 1467 were not related to our research aim. After screening, 101 studies were selected for full‐text review, including 5 studies with incomplete accessible data, 66 studies with unqualified outcome indicators, and 4 studies that did not meet the diagnostic criteria. Finally, 36 cohort studies were included (Abe et al. 2020; Abe et al. 2022; Akimoto et al. 2024; Amakasu et al. 2024; Boriesosdick et al. 2023; Choi 2024; Fukuma et al. 2023; Gubarev et al. 2025; Gwak et al. 2025; Huang et al. 2025; Jang et al. 2020; Kameyama et al. 2022; Kim et al. 2025; Lee et al. 2023; Lee et al. 2022; Lee et al. 2022; Li et al. 2022) (Figure 1; Matsushita et al. 2019; Nishioka et al. 2022; Nozoe et al. 2024; Nozoe et al. 2021; Nozoe et al. 2022; Nozoe et al. 2019; Oge et al. 2025; Ogino et al. 2024; Ohashi et al. 2025; Ohyama et al. 2020; Pinho et al. 2024; Ravera et al. 2024; Sato et al. 2025; Shiraishi et al. 2024; Tutal Gürsoy et al. 2023; Yamasaki et al. 2023; Yoshimura et al. 2019; Yoshimura et al. 2025; Zhang et al. 2025).

**FIGURE 1:**
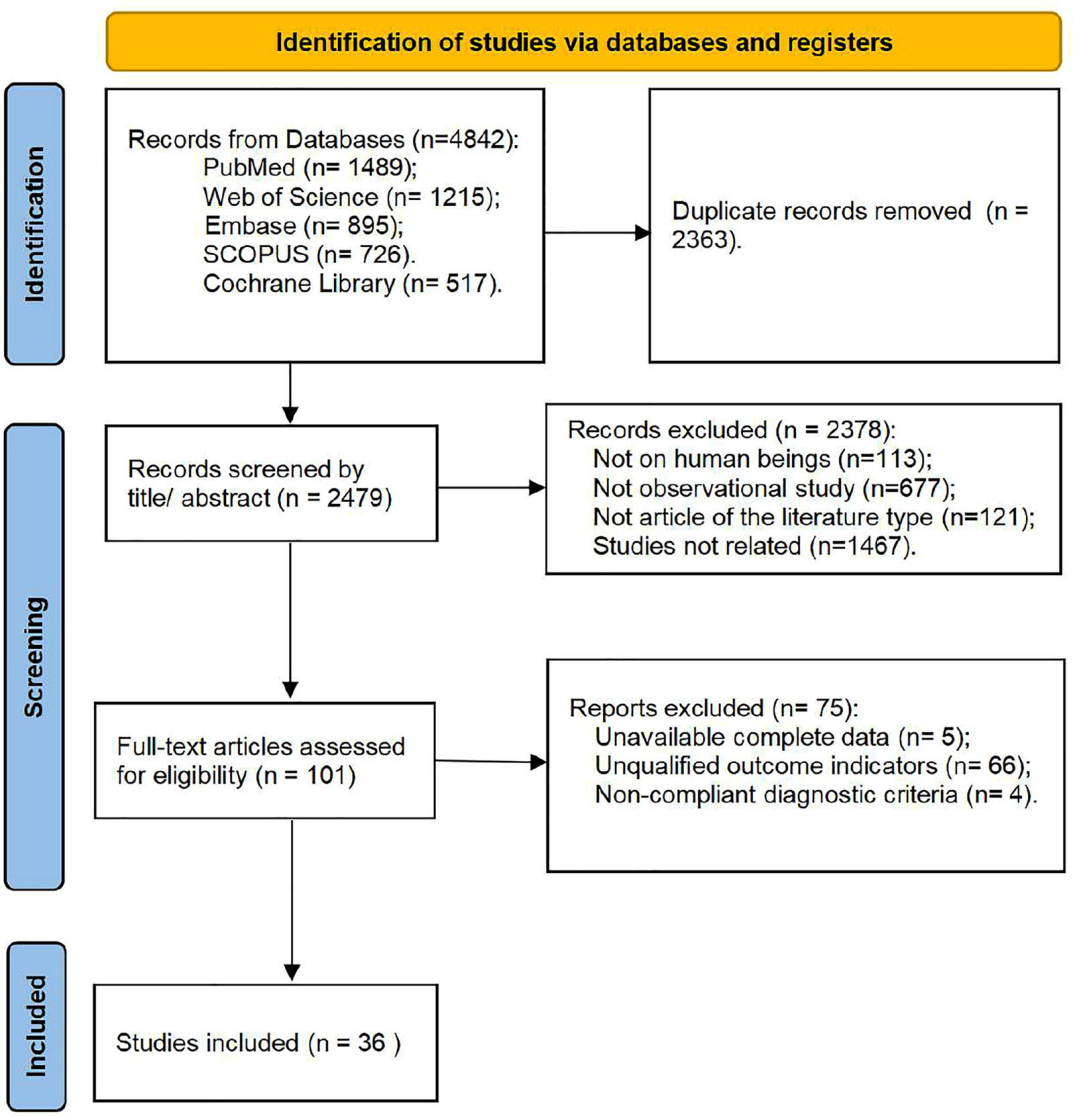
Flow chart of study selection process.

### 3.2 Study Characteristics

A total 9265 stroke patients were included. The studies were conducted in China, South Korea, Japan, Turkey, Italy, and Germany. Regarding stroke types, one study focused on hemorrhagic stroke, 16 on ischemic stroke, and 19 on both hemorrhagic and ischemic stroke. The diagnostic criteria for sarcopenia were diverse. Some studies adopted widely recognized diagnostic criteria, such as the AWGS and the EWGSOP, while others used indicators including skeletal muscle mass index (SMI), temporal muscle thickness (TMT), phase angle of bio‐impedance analysis (BIA), and Strength, Assistance with walking, Rise from a chair, Climb stairs, and Falls (SARC‐F). Among the included studies, 22 reported the mRS as the outcome indicator for global function, with follow‐up time points including discharge, 3 months, 6 months, and 1 year. Nine studies reported FIM‐motor as the outcome indicator for motor function, with all follow‐up conducted at discharge. Seven studies reported post‐stroke cognitive outcomes, of which six used FIM‐cognition and one used the MoCA as cognitive function outcome indicators, with all follow‐up performed at discharge. In addition, five studies reported post‐stroke swallowing outcomes. Among these, one used indwelling nasogastric tube, three used FOIS (< 5), and one used WST (≥ 3) as the criterion for swallowing dysfunction. The follow‐up time points of these studies included discharge, 48 h, 5 days, 2 weeks, and 3 months. The main features of the included studies are presented in Table 1.

**TABLE 1:** Study characteristics.

Study	Country	Study type	No. of sarcopenia/ non‐sarcopenia	Age	Male (%)	Stroke type	Assessment of sarcopenia	Outcomes	Follow‐up duration	NOS scores
Zhang et al. (2025)	China	Cohort	66/162	/	50	Ischemic	AWGS	①	3 months	7
Gwak et al. (2025)	South Korea	Cohort	151/449	75.3 ± 6.1	50.5	Ischemic	TMT	①④	3 months	9
Akimoto et al. (2024)	Japan	Cohort	44/67	77 (19‐99)	64	Ischemic	AWGS	①	At discharge	8
Nozoe et al. (2024)	Japan	Cohort	70/177	73 (65‐78)	66.8	Mixed	AWGS	①	3 months	8
Tutal Gürsoy et al. (2023)	Turkey	Cohort	147 in total	67.64 ± 13.38	49	Ischemic	TMT	①	3 months	9
Lee et al. (2023)	South Korea	Cohort	171/479	/	61.7	Ischemic	AWGS	①	3 months	9
Lee 2022 (Lee et al. 2022)	South Korea	Cohort	48/520	65.5 ± 12.6	64.6	Ischemic	AWGS	①	3 months	9
Nozoe et al. (2021)	Japan	Cohort	61/263	76 (71‐82)	57.7	Mixed	SARC‐F	①	3 months	8
Ravera et al. (2024)	Italy	Cohort	197/94	73.0 ± 13.0	54	Ischemic	TMT	①	3 months	8
Oge et al. (2025)	Turkey	Cohort	297 in total	67.8 ± 14.5	54.5	Ischemic	Phase Angle of BIA	①	1 year	9
Nozoe et al. (2022)	Japan	Cohort	59/252	76 (70‐82)	57.4	Mixed	SARC‐F	①	3 months	7
Abe et al. (2020)	Japan	Cohort	32/75	76.0 ± 10.4	66.4	Ischemic	SMI	①	At discharge	8
Nozoe et al. (2019)	Japan	Cohort	27/125	76 (71‐82)	53.3	Mixed	SARC‐F	①	3 months	8
Ohyama et al. (2020)	Japan	Cohort	101/63	/	65.9	Ischemic	SMI	①	At discharge	8
Jang et al. (2020)	South Korea	Cohort	81/113	64.3 ± 13.0	59.3	Mixed	Handgrip strength	①	6 months	9
Boriesosdick et al. (2023)	Germany	Cohort	189 in total	74.2 ± 13.2	45	Ischemic	Masseter muscle area	①	3 months	9
Kim et al. (2025)	South Korea	Cohort	26/73	/	75.8	Ischemic	AWGS	①	3 months	9
Huang et al. (2025)	China	Cohort	467 in total	65.37 ± 10.46	63.6	Ischemic	TMT	①	6 months	9
Amakasu et al. (2024)	Japan	Cohort	148/57	80 (74‐87)	50.7	Mixed	Phase Angle of BIA	①	At discharge	6
Gubarev et al. (2025)	Germany	Cohort	210/112	77 (66‐83)	57.5	Hemorrhagic	TMT	①	3 months	8
Sato et al. (2025)	Japan	Cohort	119/326	75 (66‐83)	62.2	Mixed	Calf circumference	①	1 year	7
Lee et al. (2022)	South Korea	Cohort	48/520	65.5 ± 12.6	64.6	Ischemic	AWGS	①	3 months	9
Shiraishi et al. (2024)	Japan	Cohort	58/60	78.8 ± 8.1	49	Mixed	AWGS	②	At discharge	8
Ogino et al. (2024)	Japan	Cohort	265/191	80 (71‐87)	51.1	Mixed	AWGS	②	At discharge	9
Nishioka et al. (2022)	Japan	Cohort	153/40	/	57.2	Mixed	AWGS	②③	At discharge	8
Yamasaki et al. (2023)	Japan	Cohort	39/34	73 (61‐81)	46	Mixed	AWGS	②③	At discharge	8
Matsushita et al. (2019)	Japan	Cohort	129/138	72.5 ±13.2	56.2	Mixed	EWGSOP	②③	At discharge	8
Yoshimura et al. (2019)	Japan	Cohort	402/393	74.9 ±13.2	40.8	Mixed	EWGSOP	②③	At discharge	8
Kameyama et al. (2022)	Japan	Cohort	163/120	76.8 ± 7.1	56.2	Mixed	EWGSOP	②③	At discharge	8
Yoshimura et al. (2025)	Japan	Cohort	621/459	75.6 ± 9.3	54.1	Mixed	Phase Angle of BIA	②	At discharge	6
Abe et al. (2022)	Japan	Cohort	21/158	73.9 ± 12.6	69.8	Mixed	SMI	②③	At discharge	9
Choi (2024)	South Korea	Cohort	29/21	75.0 ± 6.8	36	Mixed	AWGS	③	At discharge	7
Fukuma et al. (2023)	Japan	Cohort	119/204	/	63.5	Mixed	AWGS	④	2 weeks	8
Ohashi et al. (2025)	Japan	Cohort	302 in total	69.4 ± 13.8	67.5	Mixed	SMI	④	At discharge	7
Pinho et al. (2024)	Germany	Cohort	137 in total	/	56.9	Ischemic	SMI	④	5 days	6
Li et al. (2022)	China	Cohort	131/134	/	62.6	Ischemic	TMT	④	48 h	7

*Note*: ① Modified Rankin Scale (mRS); ② Functional Independence Measure‐motor (FIM‐motor); ③ Functional Independence Measure‐cognition (FIM‐cognition) or Montreal Cognitive Assessment (MoCA); ④ dysphagia assessment.

Abbreviations: AWGS, Asian Working Group for Sarcopenia; BIA, bio‐impedance analysis; EWGSOP, European Working Group on Sarcopenia in Older People; NOS, Newcastle–Ottawa Scale; SARC‐F, Strength, Assistance with walking, Rise from a chair, Climb stairs, and Falls; SMI, Skeletal Muscle Index; TMT, temporal muscle thickness.

### 3.3 Methodological Quality Assessment

All included studies achieved a NOS score of 6 points or higher (Table 1). Among them, three studies with a score of 6 were regarded as moderate‐quality research, while the remaining 33 were high‐quality studies. The main reasons for score deductions in the NOS assessment were as follows: baseline differences in outcome indicators were not controlled in 14 studies, confounding factors were not fully adjusted in five studies, the follow‐up duration failed to reach the required length in six studies, and the loss‐to‐follow‐up rate was excessively high in seven studies (Table S2).

### 3.4 Primary Outcomes

#### 3.4.1 The Impact of Sarcopenia on Global Functional Outcomes in Stroke Assessed by the mRS

Our primary outcome measure was the mRS, which reflects the overall functional recovery level of stroke patients. A total of 22 studies met the inclusion criteria for the primary outcome (Abe et al. 2020; Akimoto et al. 2024; Amakasu et al. 2024; Boriesosdick et al. 2023; Gubarev et al. 2025; Gwak et al. 2025; Huang et al. 2025; Jang et al. 2020; Kim et al. 2025; Lee et al. 2023; Lee et al. 2022; Lee et al. 2022; Nozoe et al. 2024; Nozoe et al. 2021; Nozoe et al. 2022; Nozoe et al. 2019; Oge et al. 2025; Ohyama et al. 2020; Ravera et al. 2024; Sato et al. 2025; Tutal Gürsoy et al. 2023; Zhang et al. 2025). The results of the meta‐analysis indicated that stroke patients complicated with sarcopenia had a significantly higher risk of poor functional prognosis compared with non‐sarcopenic patients (OR = 2.43, 95% CI: 1.95 to 3.03, *p* < 0.001, Figure 2). These findings suggest that sarcopenia may serve as a critical contributor to long‐term functional impairment in stroke. Specifically, stroke patients with sarcopenia were 2.43 times more likely to experience moderate‐to‐severe functional dependence than those without sarcopenia, such as inability to walk independently and requirement for assistance with activities of daily living. In addition, the results revealed high heterogeneity across the included studies (*I* ^2^ = 70%, *p* < 0.1), and thus a random‐effects model was employed for data pooling.

**FIGURE 2:**
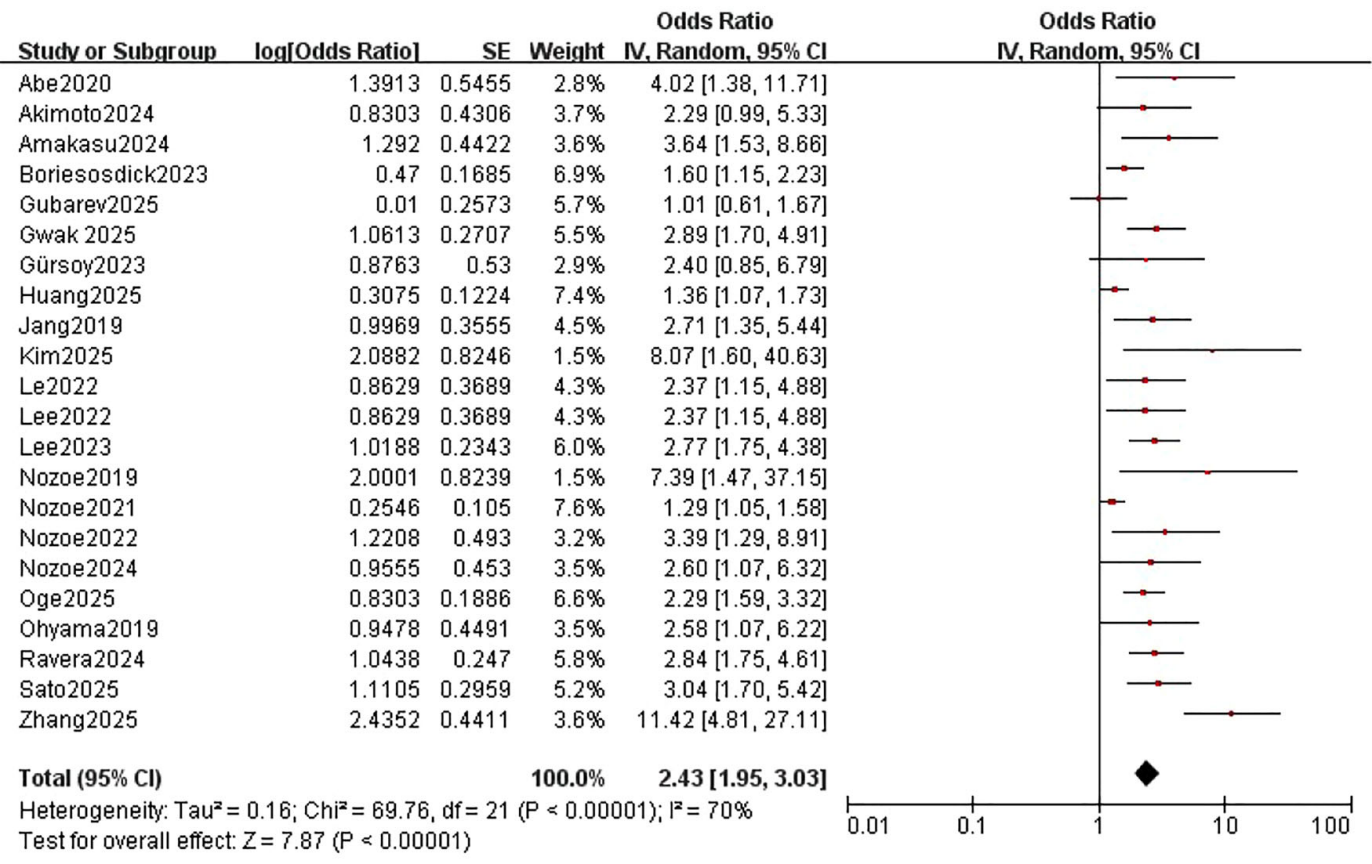
Forest plots for the meta‐analyses of sarcopenia and poor functional outcome assessed by mRS in patients with stroke.

#### 3.4.2 Subgroup Analysis

To further explore the sources of heterogeneity, subgroup analyses were performed according to the patient populations, cut‐off value of mRS for poor functional outcomes, stroke type, follow‐up duration, and diagnostic criteria for sarcopenia (Table 2).

**TABLE 2:** Subgroup analyses of sarcopenia and poor functional outcome assessed by mRS in patients with stroke.

		Heterogeneity		Meta‐analysis		Heterogeneity between subgroup	
Subgroup	No. of studies	*I* ^2^	*p* value	OR(95% CI)	*p* value	*I* ^2^	*p* value
Patient populations							
East Asian	17 (Zhang et al. 2025; Gwak et al. 2025; Akimoto et al. 2024; Nozoe et al. 2024; Lee et al. 2023; Lee et al. 2022; Nozoe et al. 2021; Nozoe et al. 2022; Abe et al. 2020; Nozoe et al. 2019; Ohyama et al. 2020; Jang et al. 2020; Kim et al. 2025; Huang et al. 2025; Amakasu et al. 2024; Sato et al. 2025; Lee et al. 2022)	73%	<0.001	2.75(2.07–3.66)	<0.001	65.5%	0.09
Non‐East Asian	5 (Tutal Gürsoy et al. 2023; Ravera et al. 2024; Oge et al. 2025; Boriesosdick et al. 2023; Gubarev et al. 2025)	63%	0.03	1.86(1.31–2.64)	<0.001		
Cut‐off value of mRS for poor functional outcome							
mRS ≥ 2	4 (Lee et al. 2022; Oge et al. 2025; Kim et al. 2025; Lee et al. 2022)	0%	0.53	2.42(1.80–3.25)	<0.001	0%	0.93
mRS ≥ 3	12 (Zhang et al. 2025; Gwak et al. 2025; Akimoto et al. 2024; Nozoe et al. 2024; Tutal Gürsoy et al. 2023; Ohyama et al. 2020; Jang et al. 2020; Boriesosdick et al. 2023; Huang et al. 2025; Amakasu et al. 2024; Gubarev et al. 2025; Sato et al. 2025)	73%	<0.001	2.37(1.72–3.27)	<0.001		
mRS ≥ 4	6 (Lee et al. 2023; Nozoe et al. 2021; Ravera et al. 2024; Nozoe et al. 2022; Abe et al. 2020; Nozoe et al. 2019)	78%	<0.001	2.66(1.59–4.45)	<0.001		
Follow‐up duration							
At discharge	4 (Akimoto et al. 2024; Abe et al. 2020; Ohyama et al. 2020; Amakasu et al. 2024)	0%	0.81	2.96(1.89–4.66)	<0.001	0%	0.66
3 months	14 (Zhang et al. 2025; Gwak et al. 2025; Nozoe et al. 2024; Tutal Gürsoy et al. 2023; Lee et al. 2023; Lee et al. 2022; Nozoe et al. 2021; Ravera et al. 2024; Nozoe et al. 2022; Nozoe et al. 2019; Boriesosdick et al. 2023; Kim et al. 2025; Gubarev et al. 2025; Lee et al. 2022)	75%	<0.001	2.51(1.83–3.44)	<0.001		
6 months	2 (Jang et al. 2020; Huang et al. 2025)	70%	0.07	1.77(0.92–3.42)	0.09		
12 months	2 (Oge et al. 2025; Sato et al. 2025)	0%	0.42	2.49(1.82–3.40)	<0.001		
Stroke type							
Ischemic	14 (Zhang et al. 2025; Gwak et al. 2025; Akimoto et al. 2024; Tutal Gürsoy et al. 2023; Lee et al. 2023; Lee et al. 2022; Ravera et al. 2024; Oge et al. 2025; Abe et al. 2020; Ohyama et al. 2020; Boriesosdick et al. 2023; Kim et al. 2025; Huang et al. 2025; Lee et al. 2022)	67%	<0.001	2.56(1.96–3.33)	<0.001	81.8%	0.004
Hemorrhagic	1 (Gubarev et al. 2025)	/	/	1.01(0.61–1.67)	0.97		
Mixed	7 (Nozoe et al. 2024; Nozoe et al. 2021; Nozoe et al. 2022; Nozoe et al. 2019; Jang et al. 2020; Amakasu et al. 2024; Sato et al. 2025)	72%	0.002	2.66(1.64–4.32)	<0.001		
Assessment of sarcopenia							
AWGS	7 (Zhang et al. 2025; Akimoto et al. 2024; Nozoe et al. 2024; Lee et al. 2023; Lee et al. 2022; Kim et al. 2025; Lee et al. 2022)	50%	0.06	3.27(2.14–4.98)	<0.001	0%	0.54
TMT	5 (Gwak et al. 2025; Tutal Gürsoy et al. 2023; Ravera et al. 2024; Huang et al. 2025; Gubarev et al. 2025)	75%	0.003	1.85(1.20–2.85)	0.003		
SARC‐F	3 (Nozoe et al. 2021; Nozoe et al. 2022; Nozoe et al. 2019)	75%	0.02	2.56(0.94–6.95)	0.07		
Phase angle of BIA	2 (Oge et al. 2025; Amakasu et al. 2024)	0%	0.34	2.46(1.75–3.46)	<0.001		
SMI	2 (Abe et al. 2020; Ohyama et al. 2020)	0%	0.53	3.09(1.56–6.09)	0.001		
others	3 (Jang et al. 2020; Boriesosdick et al. 2023; Sato et al. 2025)	55%	0.11	2.21(1.41–3.46)	<0.001		

Abbreviations: AWGS, Asian Working Group for Sarcopenia; mRS, Modified Rankin Scale; TMT, temporal muscle thickness.

SARC‐F, Strength, Assistance with walking, Rise from a chair, Climb stairs, and Falls; BIA, bio‐impedance analysis; SMI, Skeletal Muscle Index.

When stratified by patient populations, subgroup analysis was performed comparing East Asian studies and non‐East Asian studies. The results showed that sarcopenia was associated with a significantly increased risk of poor functional outcome in both East Asian and non‐East Asian populations. However, high heterogeneity remained within both subgroups (both *I* ^2^ > 50%, *p* < 0.1), and the between‐subgroup heterogeneity was also substantial (*I* ^2^ > 50%, *p* < 0.1).

When the meta‐analysis was stratified by the cut‐off value of mRS, the results showed that the within‐subgroup heterogeneity decreased significantly when mRS ≥ 2 was defined as the threshold for poor outcomes (*I* ^2^ = 0%, *p* = 0.53). In contrast, substantial heterogeneity persisted in the subgroups using mRS ≥ 3 and mRS ≥ 4 as the respective cut‐offs (*I* ^2^ > 50%, *p* < 0.001).

For subgroup analysis based on follow‐up duration, heterogeneity was markedly reduced in the subgroups with follow‐up assessments conducted at discharge and at 12 months post‐stroke (both *I* ^2^ = 0%, *p* > 0.1). Conversely, high heterogeneity was observed in the subgroups with follow‐up time points at 3 months and 6 months (both *I* ^2^ > 50%, *p* < 0.1).

When stratified by stroke type, the results indicated significant subgroup heterogeneity across ischemic stroke, hemorrhagic stroke, and mixed‐type stroke subgroups (*I* ^2^ = 81.8%, *p* = 0.004).

Regarding subgroup stratification by diagnostic criteria for sarcopenia, the findings revealed low heterogeneity in the subgroups where sarcopenia was diagnosed based on the phase angle of BIA or SMI (both *I* ^2^ = 0%, *p* > 0.1). In comparison, high heterogeneity was noted in the subgroups adopting diagnostic criteria such as the AWGS, TMT, SARC‐F questionnaire, and other alternative approaches (all *I* ^2^ ≥ 50%, *p* < 0.1).

### 3.5 Second Outcome

#### 3.5.1 The Impact of Sarcopenia on Motor Function After Stroke

The FIM‐motor was adopted as the assessment tool to evaluate the effect of sarcopenia on post‐stroke motor function, with nine studies included (Abe et al. 2022; Kameyama et al. 2022; Matsushita et al. 2019; Nishioka et al. 2022; Ogino et al. 2024; Shiraishi et al. 2024; Yamasaki et al. 2023; Yoshimura et al. 2019; Yoshimura et al. 2025). The findings demonstrated that sarcopenic patients exhibited markedly lower FIM‐motor scores after stroke than their non‐sarcopenic counterparts (SMD = −0.94, 95% CI: −1.11 to −0.77, *p* < 0.001, Figure 3), indicating poorer motor outcomes associated with sarcopenia.

**FIGURE 3:**
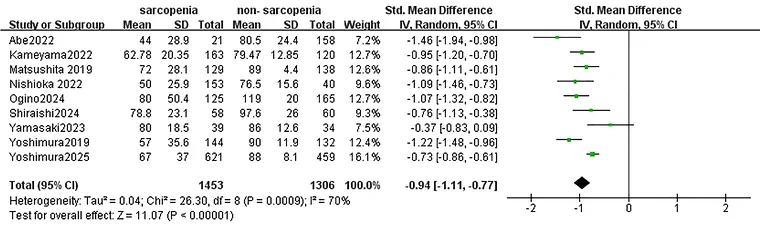
Forest plot of meta‐analysis on FIM‐motor scores in sarcopenia and non‐sarcopenia of patients with stroke.

#### 3.5.2 The Impact of Sarcopenia on Cognitive Function After Stroke

A total of seven studies were included in the meta‐analysis investigating the relationship between sarcopenia and post‐stroke cognitive function. Among these, six studies (Abe et al. 2022; Kameyama et al. 2022; Matsushita et al. 2019; Nishioka et al. 2022; Yamasaki et al. 2023; Yoshimura et al. 2019) employed the FIM‐cognition as the assessment instrument, while one study (Choi 2024) adopted the MoCA. The results showed that sarcopenic patients exhibited lower post‐stroke cognitive scores relative to non‐sarcopenic patients (SMD = −0.95, 95% CI: −1.22 to −0.69, *p* < 0.001, Figure 4), suggesting impaired cognitive function in the presence of sarcopenia.

**FIGURE 4:**
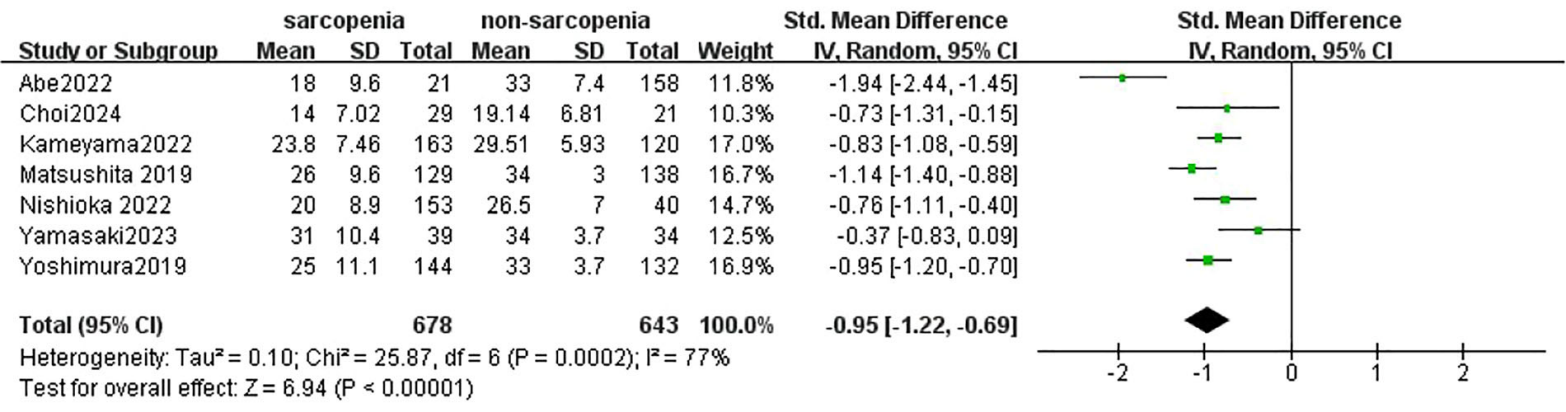
Forest plot of meta‐analysis on FIM‐cognition scores in sarcopenia and non‐sarcopenia of patients with stroke.

#### 3.5.3 The Impact of Sarcopenia on Swallowing Function After Stroke

Six studies were incorporated into the meta‐analysis exploring the influence of sarcopenia on swallowing function after stroke. The assessment criteria for swallowing dysfunction were defined as nasogastric tube placement (Gwak et al. 2025), FOIS score < 5 (Fukuma et al. 2023; Ohashi et al. 2025; Pinho et al. 2024), and water swallow test grade ≥ 3 (Li et al. 2022). The results exhibited a trend toward a higher risk of swallowing dysfunction relative to those without sarcopenia, though this association did not reach statistical significance (OR = 1.45, 95% CI: 0.94 to 2.25, *p* = 0.09, Figure 5).

**FIGURE 5:**
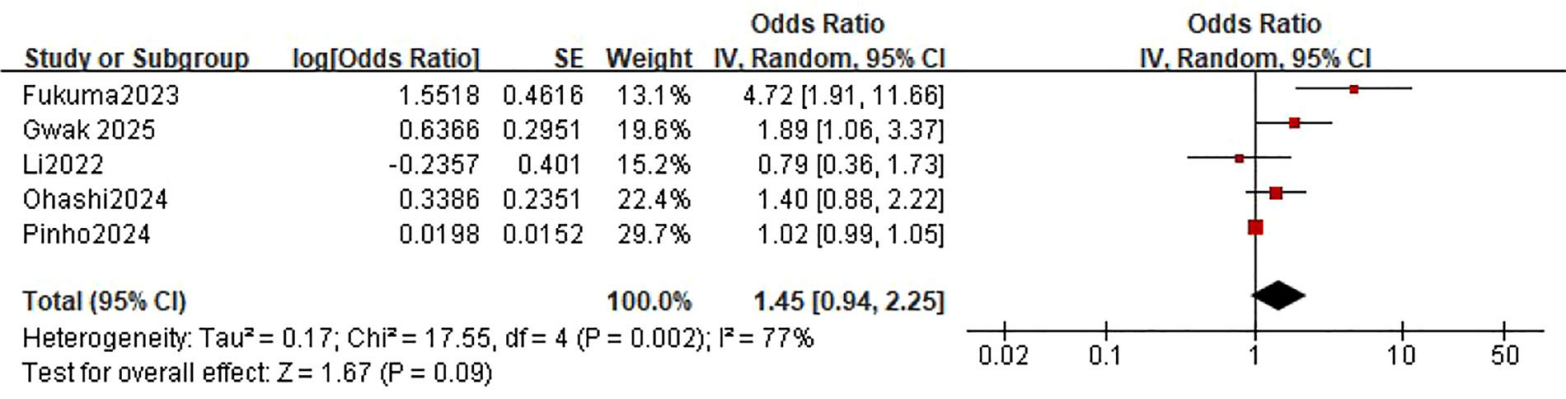
Forest plots for the meta‐analyses of sarcopenia and dysphagia in patients with stroke.

### 3.6 Publication Bias

Publication bias was assessed for the primary outcome of our meta‐analysis: the risk of poor functional outcomes associated with sarcopenia in stroke patients, as defined by the mRS. Visual inspection of the funnel plot revealed an asymmetric distribution, and the results of Egger's test further confirmed the presence of significant publication bias (*z* = 4.57, *p* < 0.001). This finding might be attributable to the effects of heterogeneity or small sample sizes across the included studies. To account for this bias, we performed the trim‐and‐fill method. After imputing seven hypothetical missing studies, the pooled results of the meta‐analysis remained consistent with the original findings (OR = 2.03, 95% CI: 1.60 to 2.56, *p* < 0.001, Figure 6). These results indicated that despite the existence of publication bias, the robustness of our primary conclusion remained unaffected.

**FIGURE 6:**
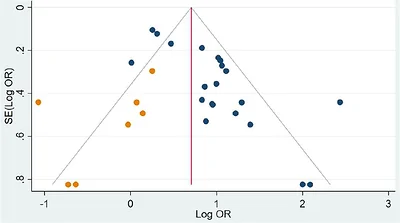
Funnel plots and trim‐and‐fill method on sarcopenia and poor functional outcome assessed by mRS in patients with stroke.

### 3.7 Sensitivity Analysis

Sensitivity analysis was conducted using the one‐study removal method. The results indicated that the direction and magnitude of the meta‐analysis findings did not change significantly after the sequential exclusion of any individual study. The conclusions remained consistent with the original results, thus confirming the stability of our findings (Table S3).

## 4 Discussion

To our knowledge, this study is the first meta‐analysis to comprehensively incorporate multi‐dimensional prognostic outcomes of stroke. We included 36 cohort studies with substantial stroke patient samples to conduct a systematic evaluation of the link between sarcopenia and multi‐dimensional functional outcomes after stroke, thereby addressing the limitation of incomplete outcome assessment in previous meta‐analyses or systematic reviews. The results demonstrated that stroke patients complicated with sarcopenia had a significantly increased risk of poor overall functional prognosis and their motor and cognitive functions were markedly worse than those of non‐sarcopenic patients, whereas a higher risk of swallowing dysfunction only showed a non‐significant trend. Subgroup analyses further identified the potential sources of heterogeneity, while sensitivity analyses and trim‐and‐fill method correction confirmed the robustness of the study conclusions. These findings indicate that sarcopenia is an important risk factor for poor long‐term multi‐dimensional functional prognosis in stroke patients, providing critical evidence‐based support for clinical prognostic evaluation and intervention.

### 4.1 The Association Between Sarcopenia and Overall Functional Prognosis of Stroke

As an internationally recognized core tool for evaluating stroke functional prognosis, the mRS quantifies patients' independent living ability, activity limitation, and disability status through a 0–6 grading system. A score of mRS ≥ 3 is generally defined as poor functional prognosis, corresponding to patients who require partial or complete assistance from others to perform daily activities, such as walking, dressing, washing, which objectively reflects unsatisfactory overall functional recovery (Kimura et al. 2026). Findings from the present study revealed that stroke patients with sarcopenia were 2.43 times more likely to experience poor neurological functional outcomes than those without sarcopenia, which is consistent with the conclusions of previous studies (Li et al. 2023).

The negative impact of sarcopenia on the overall function of stroke patients may stem from the synergistic effect of multiple mechanisms. First, central nervous system injury caused by stroke directly disrupts motor nerve pathways, and the pre‐existing skeletal muscle mass and strength deficit in sarcopenic patients further weakens the material basis for motor function recovery, leading to a significant decline in patients' ability to walk independently and perform daily activities, and ultimately resulting in elevated mRS scores (Chen et al. 2025; Sato et al. 2024). Second, sarcopenia is often accompanied by increased levels of inflammatory factors and the inflammatory response is an important factor that exacerbates post‐stroke neuronal apoptosis and hinders neural repair (Araújo et al. 2025). The combined effect of these two factors aggravates overall functional impairment, making it more difficult for patients to achieve the functional status corresponding to low mRS grades. In addition, sarcopenic patients often have poor nutritional status, and insufficient protein intake not only affects muscle repair but also may lead to neurotransmitter synthesis disorders, further impairing functional recovery and increasing the risk of adverse outcomes (Sato et al. 2022).

The primary outcome of this study showed high heterogeneity, and subgroup analyses identified potential sources. We explored the impact of patient populations on heterogeneity by comparing East Asian and non‐East Asian cohorts. Sarcopenia was associated with a significantly increased risk of poor functional outcome in both populations, but the effect size was larger in East Asian studies. Substantial heterogeneity remained within both subgroups. These findings suggest that while sarcopenia is a consistent prognostic factor across different geographic populations, the magnitude of its impact may vary. Possible explanations include differences in genetic background, lifestyle, nutritional status, and healthcare systems between East Asian and non‐East Asian countries. However, the limited number of non‐East Asian studies with relatively small sample sizes may have contributed to the observed differences. Future multi‐ethnic studies are needed to confirm these findings.

When mRS ≥ 2 was used as the poor outcome criterion, heterogeneity decreased significantly. This was likely because the definition of mRS ≥ 2 is broader. It encompasses patients with mild functional impairment and facilitates consensus among research teams, thus minimizing assessment‐related biases. In contrast, mRS ≥ 3 and mRS ≥ 4 indicate moderate‐to‐severe impairment. They exhibited high heterogeneity because their evaluation criteria are detail‐dependent and subject to assessor subjectivity.

Regarding follow‐up duration, low heterogeneity was observed at discharge and 12 months post‐stroke. These time points correspond to stable acute‐phase status and long‐term functional stabilization, respectively. In contrast, high heterogeneity existed at the 3‐ to 6‐month follow‐up interval, which represents a critical rehabilitation period with variable intervention strategies and patient compliance across different studies (Zhou et al. 2025).

In addition, high subgroup heterogeneity was also noted among ischemic, hemorrhagic, and mixed‐type stroke subgroups, possibly attributable to differences in pathological mechanisms. For example, cerebral edema and increased intracranial pressure in hemorrhagic stroke cause more significant acute damage to neurological function, while reperfusion injury and inflammatory response in ischemic stroke last longer, which may lead to varying degrees of impact of sarcopenia (Zhong et al. 2026).

Diverse sarcopenia diagnostic criteria also contributed to heterogeneity, consistent with previous findings (Yan et al. 2025). Low heterogeneity was seen with BIA‐derived phase angle and SMI, which rely on objective skeletal muscle mass measurement. In contrast, high heterogeneity occurred with the AWGS and TMT. The AWGS incorporates subjective or functional indicators, while the TMT is not a standard diagnostic method and is applied with variable cut‐off values across studies. Despite these limitations regarding TMT as a diagnostic tool, recent evidence supports its prognostic value. Ravera et al. (2024) demonstrated that TMT measured on baseline CT scans is an independent predictor of survival and functional outcome in acute ischemic stroke patients undergoing reperfusion therapies, with higher TMT values associated with reduced mortality. This suggests that while TMT may not be a standardized diagnostic criterion for sarcopenia, it remains a readily available and clinically useful prognostic marker in acute stroke settings.

These results suggest that future studies should adopt unified diagnostic criteria for sarcopenia and outcome assessment methods to reduce heterogeneity and improve the comparability of research results. Meanwhile, more targeted intervention strategies need to be developed for patients in different subgroups. Future multi‐ethnic studies are also needed to confirm these findings across diverse populations.

### 4.2 The Impact of Sarcopenia on Motor, Cognitive, and Swallowing Function Outcomes After Stroke

In terms of motor outcomes, this study found that patients with sarcopenia had significantly lower FIM‐motor scores than non‐sarcopenic counterparts, indicating a close association between sarcopenia and post‐stroke motor dysfunction. The FIM‐motor scale covers core motor domains including self‐care, transfer, and ambulation. A reduced score on this scale reflects a marked decline in patients' motor independence. Impaired muscle secretory function and decreased myokine levels in sarcopenic patients impede post‐stroke neural regeneration and synaptic remodeling, thereby hindering motor function recovery (Han et al. 2025). Additionally, muscle atrophy and reduced muscle strength in these patients increase the difficulty of rehabilitation training, lower treatment compliance, and further exacerbate motor impairment. Guerrini et al. (2025) prospectively followed 87 subacute stroke patients undergoing a 6‐week rehabilitation program and found that patients with sarcopenia diagnosed according to EWGSOP2 criteria exhibited significantly poorer gains in functional independence and ambulation compared with non‐sarcopenic patients, despite receiving comparable rehabilitation interventions. Notably, this is one of the studies to validate the prognostic role of sarcopenia in a non‐Asian population, thereby extending the generalizability of our findings beyond East Asian cohorts.

To our knowledge, this study is the first to confirm a significant correlation between sarcopenia and post‐stroke cognitive decline through meta‐analysis, expanding the current understanding of the detrimental effects of sarcopenia. The potential mechanisms underlying the effect of sarcopenia on cognitive function may include two aspects. Muscle tissue can reduce the risk of cerebrovascular disease progression by regulating insulin sensitivity and mitigating inflammatory responses. However, the insulin resistance and chronic inflammatory state in sarcopenic patients may exacerbate insufficient cerebral blood perfusion, impairing the function of cognition‐related brain regions such as the hippocampus and frontal lobe (Farhana et al. 2026; Oudbier et al. 2022). Besides, myokines such as irisin and BDNF are essential for neuronal survival and cognitive function maintenance. Reduced myokine secretion caused by sarcopenia may directly impair cognitive functions including spatial learning and memory (Cui et al. 2025). Furthermore, decreased activity capacity and reduced social participation in stroke patients with sarcopenia may also indirectly compromise cognitive function maintenance.

Additionally, this study indicated that stroke patients with sarcopenia tended to have an elevated risk of swallowing dysfunction, a trend that may be closely linked to the physiological regulatory mechanisms of swallowing function. The completion of swallowing requires the coordinated contraction of multiple skeletal muscle groups, such as the lingual, pharyngeal, and laryngeal muscles. Systemic skeletal muscle mass loss and reduced muscle strength induced by sarcopenia inevitably impair the function of swallowing‐related muscles. Moreover, central nervous system injury after stroke may lead to abnormal regulation of the swallowing reflex. Sarcopenia further weakens the compensatory capacity of swallowing muscles, which may potentially elevate the risk of dysphagia and aspiration pneumonia (Fukuma et al. 2023). However, this non‐significant trend may be attributed to the limited number of included studies, and future investigations with a larger volume of eligible literature are warranted to enhance the reliability of the results.

### 4.3 Strengths, Limitations, and Clinical Implications of the Study

This study comprehensively evaluated the association between sarcopenia and multi‐dimensional functional prognosis of stroke, including global function, motor function, cognitive function, and swallowing function. A large number of studies with substantial sample sizes were included, ensuring high evidence quality. What's more, comprehensive subgroup analyses were performed to identify potential sources of heterogeneity, providing more precise references for clinical practice. Additionally, sensitivity analysis and the trim‐and‐fill method were employed to correct publication bias, confirming the robustness of the study conclusions.

This study also has certain limitations. Most of the included studies were from Asia, which may lead to regional differences in the results. Some studies did not report in detail the adjustment of confounding factors such as nutritional status, comorbidities and rehabilitation interventions, potentially resulting in residual confounding bias. Although subgroup analyses were conducted to explore some sources of heterogeneity, there may still be unrecognized sources of heterogeneity.

This study confirms that sarcopenia is an important risk factor for poor multi‐dimensional functional prognosis in stroke patients, suggesting that sarcopenia screening should be integrated into the routine evaluation system for stroke patients in clinical practice. For patients diagnosed with sarcopenia, individualized comprehensive intervention programs should be developed, including nutritional support, resistance training, and aerobic exercise, to increase skeletal muscle mass and improve muscle function. Meanwhile, early identification and targeted rehabilitation training for cognitive and swallowing disorders should be strengthened. In addition, emphasis should be placed on long‐term follow‐up after discharge, with dynamic monitoring of patients' muscle function and nutritional status, and timely adjustment of intervention programs to improve long‐term quality of life.

## 5 Conclusion

This study provides high‐quality evidence‐based proof that sarcopenia serves as an important risk factor for poor prognosis in stroke patients. It suggests that sarcopenia screening should be incorporated into the routine evaluation of stroke patients in clinical practice. Timely individualized intervention measures such as nutritional support and resistance training should be implemented for high‐risk populations. Meanwhile, attention should be paid to the comprehensive rehabilitation of multi‐dimensional functions to improve patients' long‐term quality of life. Future research needs to conduct more multi‐center intervention studies with unified diagnostic criteria and further explore the underlying molecular mechanisms, such as myokine‐mediated neural repair pathways and the regulation of inflammatory responses, so as to provide a theoretical basis for targeted therapy.

## Author Contributions

**Dandan Xie**: conceptualization, methodology, formal analysis, funding acquisition, Writing – original draft, supervision, Writing – review and editing. **Lu Chen**: formal analysis, investigation, writing – review and editing. **Mingming Ma**: software, data curation, validation, writing – original draft.

## Funding

The study was supported by the Traditional Chinese Medicine Science and Technology Program of Zhejiang Province, No.2026ZL0571 and the Key Cultivated Discipline of Hangzhou Municipality: Integrated Traditional Chinese and Western Medicine in Rehabilitation Medicine, No. 2025HZPY02.

## Conflicts of Interest

The authors declare no conflicts of interest.

## Supporting information




**Supplementary Material**: brb371810‐sup‐0001‐SuppMat.doc

## Data Availability

The datasets used and/or analyzed during the current study are available from the corresponding author on reasonable request.
