# Supplementary material for: Association of Sarcopenia With Global, Motor, Cognitive, and Swallowing Function Prognosis in Stroke Patients: A Systematic Review and Meta‐Analysis
Source: Brain Behav. 2026 Sep 29;16(10):e71810. doi: 10.1002/brb3.71810 (PMC13624536; doi:10.1002/brb3.71810)
Supplement: Supplementary file 1 [file BRB3-16-e71810-s001.doc]

[**Supplementary**](http://www.baidu.com/link?url=MVyP9k1DM2BYe_09FfPQKbRbJuDFVcAG9RqDJfdCGCaS2YYwTlgba3AjNTSX32HNSPXNuxCgIKHASdMsRXzeOzUhyEXxm8YPMx5l1i9cPDC)

**Table S1. Search Strategies**

| **Databases** | **Search** | **Query** | **No. of Results** |
| --- | --- | --- | --- |
| **PubMed** | #1 | "stroke"[Mesh] | 196,535 |
|  | #2 | "stroke"[Title/Abstract] OR "cerebrovascular accident"[Title/Abstract] OR "cerebrovascular disorders"[Title/Abstract] OR "cerebral infarction"[Title/Abstract] OR "cerebral hemorrhage"[Title/Abstract] OR "cerebrovascular infarction"[Title/Abstract] OR "intracranial hemorrhage" [Title/Abstract] | 402,902 |
|  | #3 | #1 OR #2 | 438,466 |
|  | #4 | "sarcopenia"[MeSH] | 14,074 |
|  | #5 | "sarcopenia"[Title/Abstract] OR "muscle loss"[Title/Abstract] OR "muscular atrophy"[Title/Abstract] OR "muscle weakness"[Title/Abstract] OR "muscle atrophy"[Title/Abstract] OR "muscle wasting"[Title/Abstract] OR "muscle mass"[Title/Abstract] OR "muscle index"[Title/Abstract] OR "sarcopenic"[Title/Abstract] OR "sarcopaenic"[Title/Abstract] OR "sarcopaenia"[Title/Abstract] | 94,301 |
|  | #6 | #4 OR #5 | 94,744 |
|  | #7 | #3 AND #6 | ***1489*** |
| **Cochrane Library** | #1 | MeSH descriptor: [stroke] explode all trees | 18,735 |
|  | #2 | ("stroke" OR "cerebrovascular accident" OR "cerebrovascular disorders" OR "cerebral infarction" OR "cerebral hemorrhage" OR "cerebrovascular infarction" OR "intracranial hemorrhage"):ti,ab,kw | 88,937 |
|  | #3 | #1 OR #2 | 89,071 |
|  | #4 | MeSH descriptor: [sarcopenia] explode all trees | 1,099 |
|  | #5 | ("sarcopenia" OR "muscle loss" OR "muscular atrophy" OR "muscle weakness" OR "muscle atrophy" OR "muscle wasting" OR "muscle mass" OR "muscle index" OR "sarcopenic" OR "sarcopaenic" OR "sarcopaenia"):ti,ab,kw | 14,466 |
|  | #6 | #4 OR #5 | 14,466 |
|  | #7 | #3 AND #6 | ***517*** |
| **Embase** | #1 | 'stroke':ab,ti | 587,038 |
|  | #2 | 'cerebrovascular accident':ab,ti | 10,188 |
|  | #3 | 'cerebrovascular disorders':ab,ti | 2,753 |
|  | #4 | 'cerebral infarction':ab,ti | 29.173 |
|  | #5 | 'cerebral hemorrhage':ab,ti | 8,878 |
|  | #6 | 'cerebrovascular infarction':ab,ti | 128 |
|  | #7 | 'intracranial hemorrhage':ab,ti | 24,976 |
|  | #8 | #1 OR #2 OR #3 OR #4 OR #5 OR #6 OR #7 | 633,483 |
|  | #9 | 'sarcopeni*':ab,ti | 34,648 |
|  | #10 | 'sarcopaeni*':ab,ti | 159 |
|  | #11 | 'muscle loss':ab,ti,kw | 5,933 |
|  | #12 | 'muscular atrophy':ab,ti,kw | 16,655 |
|  | #13 | 'muscle atrophy':ab,ti,kw | 17,736 |
|  | #14 | 'muscle weakness':ab,ti,kw | 37,347 |
|  | #15 | 'muscle wasting':ab,ti,kw | 10,596 |
|  | #16 | 'muscle mass':ab,ti,kw | 49,011 |
|  | #17 | 'muscle index':ab,ti,kw | 5,629 |
|  | #18 | #9 OR #10 OR #11 OR #12 OR #13 OR #14 OR #15 OR #16 OR #17 | 140,998 |
|  | #19 | #8 AND #18 | 2,446 |
|  | #20 | #19 AND 'article'/it | 895 |
| **Web of Science(Core Collection)** | #1 | stroke (Topic) or "cerebrovascular accident" (Topic) or "cerebrovascular disorders" (Topic) or "cerebral infarction" (Topic) or "cerebral hemorrhage" (Topic) or "cerebrovascular infarction" (Topic) or "intracranial hemorrhage" (Topic) | 299,915 |
|  | #2 | [sarcopenia (Topic) or "muscle loss" (Topic) or "muscular atrophy" (Topic) or "muscle weakness" (Topic) or "muscle atrophy" (Topic) or "muscle wasting" (Topic) or "muscle mass" (Topic) or "muscle index" (Topic) or "sarcopenic" (Topic) or "sarcopaenia" (Topic) or "sarcopaenic" (Topic)](http://webofscience-clarivate-cn-s.webvpn.hmc.edu.cn:8118/wos/woscc/summary/d63c715c-3d23-48d8-be5f-db089c07ecd0-01928c1c76/relevance/1) | 68,803 |
|  | #3 | #1 AND #2 | ***1,215*** |
| **Scopus** | #1 | TITLE-ABS-KEY ( stroke) | 618,387 |
|  | #2 | TITLE-ABS-KEY (sarcopenia) | 33,021 |
|  | #3 | #1 AND #2 | ***726*** |

**Table S2. Quality assessment of cohort study with NOS scores**

| Study | Representativeness of the exposed cohort | Selection of the non exposed cohort | Assessment  of exposure | Demonstration that outcome of interest was not present at start | Control for important factor and additional factor | Outcome assessment | Follow up long enough for outcome to occur | Adequacy of follow up | Total NOS scores |
| --- | --- | --- | --- | --- | --- | --- | --- | --- | --- |
| Zhang 2025 [17] | 1 | 1 | 1 | 0 | 2 | 1 | 1 | 0 | 7 |
| Gwak 2025 [18] | 1 | 1 | 1 | 1 | 2 | 1 | 1 | 1 | 9 |
| Akimoto 2024 [19] | 1 | 1 | 1 | 1 | 2 | 1 | 0 | 1 | 8 |
| Nozoe 2024 [20] | 1 | 1 | 1 | 1 | 2 | 1 | 1 | 0 | 8 |
| Gürsoy 2023 [21] | 1 | 1 | 1 | 1 | 2 | 1 | 1 | 1 | 9 |
| Lee 2023 [22] | 1 | 1 | 1 | 1 | 2 | 1 | 1 | 1 | 9 |
| Le 2022 [23] | 1 | 1 | 1 | 1 | 2 | 1 | 1 | 1 | 9 |
| Nozoe 2021 [24] | 1 | 1 | 1 | 0 | 2 | 1 | 1 | 1 | 8 |
| Ravera 2024 [25] | 1 | 1 | 1 | 1 | 2 | 1 | 1 | 0 | 8 |
| Oge 2025 [26] | 1 | 1 | 1 | 1 | 2 | 1 | 1 | 1 | 9 |
| Nozoe 2022 [27] | 1 | 1 | 1 | 0 | 2 | 1 | 1 | 0 | 7 |
| Abe 2020 [28] | 1 | 1 | 1 | 1 | 2 | 1 | 0 | 1 | 8 |
| Nozoe 2019 [29] | 1 | 1 | 1 | 1 | 2 | 1 | 1 | 0 | 8 |
| Ohyama 2019 [30] | 1 | 1 | 1 | 1 | 2 | 1 | 0 | 1 | 8 |
| Jang 2019 [31] | 1 | 1 | 1 | 1 | 2 | 1 | 1 | 1 | 9 |
| Boriesosdick 2023 [32] | 1 | 1 | 1 | 1 | 2 | 1 | 1 | 1 | 9 |
| Kim 2025 [33] | 1 | 1 | 1 | 1 | 2 | 1 | 1 | 1 | 9 |
| Huang 2025 [34] | 1 | 1 | 1 | 1 | 2 | 1 | 1 | 1 | 9 |
| Amakasu 2024 [35] | 1 | 1 | 0 | 0 | 2 | 1 | 0 | 1 | 6 |
| Gubarev 2025 [36] | 1 | 1 | 1 | 1 | 2 | 1 | 1 | 0 | 8 |
| Sato 2025 [37] | 1 | 1 | 1 | 0 | 2 | 1 | 1 | 0 | 7 |
| Lee 2022 [38] | 1 | 1 | 1 | 1 | 2 | 1 | 1 | 1 | 9 |
| Shiraishi 2024 [39] | 1 | 1 | 1 | 0 | 2 | 1 | 1 | 1 | 8 |
| Ogino 2024 [40] | 1 | 1 | 1 | 1 | 2 | 1 | 1 | 1 | 9 |
| Nishioka 2022 [41] | 1 | 1 | 1 | 0 | 2 | 1 | 1 | 1 | 8 |
| Yamasaki 2023 [42] | 1 | 1 | 1 | 1 | 1 | 1 | 1 | 1 | 8 |
| Matsushita 2019 [43] | 1 | 1 | 1 | 0 | 2 | 1 | 1 | 1 | 8 |
| Yoshimura 2019 [44] | 1 | 1 | 1 | 0 | 2 | 1 | 1 | 1 | 8 |
| Kameyama 2022 [45] | 1 | 1 | 1 | 0 | 2 | 1 | 1 | 1 | 8 |
| Yoshimura 2025 [46] | 1 | 1 | 1 | 0 | 0 | 1 | 1 | 1 | 6 |
| Abe 2022 [47] | 1 | 1 | 1 | 1 | 2 | 1 | 1 | 1 | 9 |
| Choi 2024 [48] | 1 | 1 | 1 | 1 | 0 | 1 | 1 | 1 | 7 |
| Fukuma 2023 [49] | 1 | 1 | 1 | 0 | 2 | 1 | 1 | 1 | 8 |
| Ohashi 2024 [50] | 1 | 1 | 1 | 0 | 1 | 1 | 1 | 1 | 7 |
| Pinho 2024 [51] | 1 | 1 | 0 | 0 | 2 | 1 | 0 | 1 | 6 |
| Li 2022 [52] | 1 | 1 | 1 | 1 | 1 | 1 | 0 | 1 | 7 |

**Table S3. Sensitivity analysis of sarcopenia and poor functional outcome assessed by mRS in patients with stroke using the method of one-study removal**

| **Study of Omission** | **Heterogeneity** | | **Meta-analysis** | |
| --- | --- | --- | --- | --- |
|  | **I^2^** | **p value** | **OR（95%CI）** | **p value** |
| Zhang 2025 [17] | 62% | ＜0.001 | 2.24 (1.83, 2.73) | ＜0.001 |
| Gwak 2025 [18] | 70% | ＜0.001 | 2.41 (1.92, 3.02) | ＜0.001 |
| Akimoto 2024 [19] | 71% | ＜0.001 | 2.44 (1.94, 3.06) | ＜0.001 |
| Nozoe 2024 [20] | 71% | ＜0.001 | 2.43 (1.93, 3.05) | ＜0.001 |
| Gürsoy 2023 [21] | 71% | ＜0.001 | 2.43 (1.94, 3.05) | ＜0.001 |
| Lee 2023 [22] | 70% | ＜0.001 | 2.41 (1.92, 3.03) | ＜0.001 |
| Le 2022 [23] | 71% | ＜0.001 | 2.44 (1.94, 3.06) | ＜0.001 |
| Nozoe 2021 [24] | 63% | ＜0.001 | 2.55 (2.03, 3.19) | ＜0.001 |
| Ravera 2024 [25] | 70% | ＜0.001 | 2.41 (1.92, 3.03) | ＜0.001 |
| Oge 2025 [26] | 71% | ＜0.001 | 2.46 (1.94, 3.11) | ＜0.001 |
| Nozoe 2022 [27] | 71% | ＜0.001 | 2.40(1.92, 3.01) | ＜0.001 |
| Abe 2020 [28] | 70% | ＜0.001 | 2.39 (1.91, 2.99) | ＜0.001 |
| Nozoe 2019 [29] | 70% | ＜0.001 | 2.38 (1.91, 2.97) | ＜0.001 |
| Ohyama 2019 [30] | 71% | ＜0.001 | 2.43 (1.94, 3.05) | ＜0.001 |
| Jang 2019 [31] | 71% | ＜0.001 | 2.42 (1.93, 3.04) | ＜0.001 |
| Boriesosdick 2023 [32] | 71% | ＜0.001 | 2.53 (1.99, 3.21) | ＜0.001 |
| Kim 2025 [33] | 70% | ＜0.001 | 2.38 (1.91, 2.96) | ＜0.001 |
| Huang 2025 [34] | 68% | ＜0.001 | 2.56 (2.02, 3.23) | ＜0.001 |
| Amakasu 2024 [35] | 70% | ＜0.001 | 2.39 (1.91, 2.99) | ＜0.001 |
| Gubarev 2025 [36] | 69% | ＜0.001 | 2.55 (2.04, 3.19) | ＜0.001 |
| Sato 2025 [37] | 70% | ＜0.001 | 2.40 (1.91, 3.01) | ＜0.001 |
| Lee 2022 [38] | 71% | ＜0.001 | 2.44 (1.94, 3.06) | ＜0.001 |
